# EUPopLink Country report - United Kingdom

**DOI:** 10.12688/openreseurope.23498.1

**Published:** 2026-04-19

**Authors:** Mike Bolt

**Affiliations:** 1 Cardiff University School of Law and Politics, Cardiff, Wales, UK

**Keywords:** Populism, Euroscepticism, United Kingdom

## Abstract

This country report provides a summary of the UK-EU relationship since the UK’s entry in 1973. The report explores populist actors in the United Kingdom, with an emphasis on these actors in the period since the global financial crisis in 2008. The report notes that there have been both left and right populist parties active in the UK in this period. It explores key populist actors (George Galloway, Jeremy Corbyn, Nigel Farage) and parties including the Respect Party, UKIP, the Brexit Party, and Reform UK. It examines the basis of these party’s euroscepticism, their broader positions on the UK-EU relationship, and how they have mobilised populism and populist ideas. It finds that populist Eurosceptic parties belonging to the Populist Radical Right (PRR) have achieved the most success and influence in the UK context. The report also considers the response of the UK’s traditional largest political parties (the Conservative Party, the Labour Party, and the Liberal Democrats) to the rise of Euroscepticism in the UK.

## 1. Introduction

The present country report was prepared as part of the EUPopLink COST Action ( Andreadis & Vasilopoulou, 2026) following the EUPopLink Theoretical Framework and guidelines ( Lorimer et al., 2026).

The UK has been described as an “awkward partner” ( George, 1998), an “outsider” ( Daddow, 2015) and “semi-detached” ( Gamble, 2018) in relation to the EU and its precursors. Adopting a longer historical lens, aspects of Britain’s political elite can be considered, what was later termed, “Eurosceptic”, ever since the UK’s accession in 1973. A referendum held in 1975 on whether the UK should stay in the EEC saw politicians from the left and right campaign to leave the EEC ( Aqui, 2020; King, 1977; Saunders, 2018; Smith, 1999). The Eurosceptic mantel was later picked up by parties in the mid 1990’s including the short-lived Referendum Party and the United Kingdom Independence Party (UKIP) ( Ford and Goodwin, 2014: 26–7). UKIP grew from humble origins to obtain significant political influence and achieve some notable electoral successes, particularly, and somewhat ironically, in European Parliament (EP) elections. The party achieved second place in 2009, with 13 MEPs ( Hayton, 2010: 29), before finishing first in the 2014 EP elections. They also achieved some success in general elections, including in 2015 where it won over 3 million votes. UKIP’s success flourished around the late 2000s onwards as its leader, Nigel Farage, fused Eurosceptic and populist concerns, utilising events such as the 2009 expenses scandal for political benefit.

For the most part, the British public has largely been sceptical of European integration since its entry, occasionally exhibiting more positive attitudes at times, such as during the early 1990s. The British public has consistently been more sceptical about the benefits of membership compared to those in the EU-8 (the original Six EEC members, plus Denmark and Ireland) (see Hobolt and Tilley, 2021). The EU’s salience notably increased during the 2000s and 2010s, reaching a fever pitch by the time of the 2016 EU membership referendum which resulted in a narrow decision to leave the EU.

There are arguably several key turning points connected to the mainstreaming of Euroscepticism in the UK and explicit pressures to exit the EU, including the decision made by the Labour government in 2004 not to apply any restrictions to migration from the A8 countries that had recently joined. Other notable, influential, factors include the 2008 financial crash, the subsequent period of austerity politics, the growing influence of UKIP as a political force and increased prominence of Nigel Farage, and relatedly, the increased division within the Conservative Party over Europe. Populist Eurosceptic parties belonging to the populist radical right (PRR) have achieved the most success and influence within the UK context.

## 2. Populist political actors

Both left and right populist parties can be observed in the UK. Left-wing populist parties include the Respect Party, led by George Galloway, and the nascent Your Party, co-founded by Jeremy Corbyn. Your Party emerged in 2025, targeting the ‘rigged’ system and calling for a “mass redistribution of wealth and power” ( Your Party, 2025), though its full policy programme is not yet clear and as such is omitted, here. PRR parties have exerted the most influence however, specifically those connected to Nigel Farage: UKIP, the Brexit Party, and Reform UK. Further political actors have employed populist discourse, styles, and strategies such as Nicola Sturgeon (SNP) and Jeremy Corbyn (as a member of the Labour Party) (see Tindall, 2022), though their parties would likely not be consistently populist in themselves.

### 2.1 The Respect Party

George Galloway’s Respect Party was a far-left populist party. It was originally founded as a single-issue party protesting the Iraq War ( Baker and Schnapper, 2015:102). Respect outlined a far-left programme including renationalisation, higher taxes for businesses and wealthy individuals, and the reversal of anti-trade union laws ( Lynch, 2007:331–2). Their success was relatively limited and localised. Galloway was elected as an MP in 2005 and 2012, while Respect also won 16 council seats in the 2006 local elections ( Baker and Schnapper, 2015:102). ^1^

### 2.2 United Kingdom Independence Party (UKIP)

UKIP’s origins lie in the Anti-Federalist League, established in the early 1990s ( Ford and Goodwin, 2014: 2). UKIP initially resided on the political fringes and was plagued by in-fighting, divisions, and amateurism. UKIP’s professionalisation was spearheaded by its leader, Nigel Farage. It gained increased prominence during the 2000s and 2010s and had an important supporting role in the 2016 referendum. UKIP’s position in that campaign was complicated and obscured by the Leave.EU group, also led by Farage. Post-2016, the party fell away after Farage’s resignation, the election of a new leader, and its subsequent implosion ( Tournier-Sol, 2021: 383–5). During UKIP’s political peak, it cultivated links with other Eurosceptic and populist parties in the EP. After the 2009 EP election, UKIP joined twelve others, including the Danish People’s Party and the True Finns, to form the “Europe of Freedom and Democracy” (EFDD) group ( Goodwin and Milazzo, 2015: 117–19). The EFDD included controversial figures such as Mario Borghezio and Francesco Speroni from the Italian Northern League (ibid.). A further one-time key UKIP partner within the EP and the EFDD was Italy’s Five Star Movement (FSM), then led by Beppe Grillo (see Carlotti, 2018).

### 2.3 The Brexit Party

The Brexit Party was established in 2019 in response to the EP election that should not have been, given the UK vote to leave the EU nearly three years prior to this. The UK was obliged to hold European elections in May 2019 following Theresa May’s failure to secure backing for her withdrawal deal. The Brexit Party, led by Farage, was quickly formed in response. It presented little in terms of policy or a manifesto, although this did not hold them back ( Tournier-Sol, 2021: 386). It won the 2019 EP election, “securing 31.6% of the vote and returning 29 MEPs to the European parliament” (ibid.). The party’s short participation in the EP because of the UK’s exit a matter of months later seemingly meant limited opportunities to forge links with other populist actors. The Brexit Party participated in the 2019 UK General Election; however, it achieved limited success, impacted by Farage’s decision to unilaterally stand-down its candidates in Conservative held seats ( Dennison, 2020: 131).

### 2.4 Reform UK

The Brexit Party was subsequently renamed Reform UK in January 2021. The party has been led by both Nigel Farage and Richard Tice (formerly of Leave.EU), with the pair alternating between positions as party chairman and party leader, respectively. Reform UK was initially concerned with constitutional issues and constitutional reform, such as the reform of the House of Lords ( Dennison, 2020: 132). They performed well in the 2024 General Election, winning 4.1 million votes ( Hayton, 2025) and returning 5 seats. The party was lockdown-sceptic earlier on its inception during the Covid-19 pandemic. It primarily focuses on immigration, multiculturalism, public service provision, cutting government spending and has opposed Net Zero measures. Today, it is a significant political force. YouGov voting intention polling in early November 2025 revealed 27% would vote Reform UK if a general election were held tomorrow ( YouGov, 2025).

## 3. Positions on ‘Europe’ and the Populism-Euroscepticism nexus

Respect’s Euroscepticism was based upon perceptions of the EU’s neoliberal, pro-business, economic agenda ( Clark et al., 2008: 520, 522), though given its limited impact, it is excluded from further substantive analysis. UKIP, the Brexit Party, and Reform UK are the primary, populist, Eurosceptic parties in the British case. These parties are all intertwined with Farage, who variously served as leader or party chairman for each. Despite some overlap, they largely operated during distinct time periods, with Farage’s involvement proving key to any success. Farage’s affiliation with UKIP ran from 1993 to 2018; the Brexit Party between 2019 and 2020; and Reform UK from 2021 to the present.

UKIP can be considered a “hard” Eurosceptic party, as it has been “utterly opposed” to continued EU membership since its inception ( Bale, 2018: 268). The party was “sceptical before it was populist” (ibid.: 265). There seemed to be little change or evolution in its attitude towards the EU, though its overall policy programme and agenda did expand over time to encompass a wider range of issues and policies. Interestingly, the party’s views on participation in EU institutions also softened. Alan Sked initially argued UKIP should reject any seats it might win in the EP to try and bring about an EU constitutional crisis ( Ford and Goodwin, 2014: 25; Usherwood, 2019: 1211), although it subsequently took up its EP seats. Farage’s preferred vision of Europe was intergovernmental in nature, consisting of “a Europe of sovereign independent states that are friends, and mates, and neighbours with each other” ( Farage, 2016).

UKIP’s Euroscepticism revolved around the themes of democracy, freedom, and the limits imposed by the EU on Britain’s global aspirations (see Tournier-Sol, 2015). The party’s democratic critique of the EU was premised on the institution’s supposed lack of democratic legitimacy and the lack of consent from the British public for membership - given the EU’s significant changes and evolution since the Yes vote in 1975 (ibid.:142–143). The restrictions placed on Britain’s freedom and liberty as an EU member further underpinned their opposition, with the party libertarian at its heart (ibid.). Its belief that Britain should adopt a more global orientation and look beyond the confines of a regional organisation like the EU further bolstered their Eurosceptic position (ibid.:144). As an alternative to the EU, the party often looked towards the Commonwealth as a ready-made substitute. While UKIP remained steadfast in its Euroscepticism, it later evolved via an increased focus on immigration and the successful fusing of the EU and immigration ( Lynch, 2015: 196). The party’s “withdrawalist position” remained, whilst they emphasised how the mainstream parties were unable/unwilling to apply curbs to intra-EU migration i.e. free movement ( Usherwood, 2019: 1212). UKIP adopted an increasingly populist style and discourse which characterised the mainstream parties as part of a remote Westminster bubble, far removed from reality ( Hayton, 2016:406). UKIP, in contrast, was self-styled as ‘the people’s Army’ (ibid.). Farage targeted so called career politicians and the Westminster elite, who he claimed had never had a proper job or done an honest day’s work. He positioned himself as on the side of ordinary, honest, decent, hard-working people, in opposition to political elites and multinational corporations.

Farage’s creation of the Brexit Party was interesting given that UKIP’s driving mission – leaving the EU – had already been achieved in conjunction with the Leave campaigns in 2016. Its creation appeared to stem from concerns that Brexit may be soft or watered down, with the government’s Brexit deal deemed to be a betrayal of the Leave vote by a Remain-inclined parliament ( Dennison, 2020: 129; Vasilopoulou, 2020: 84). The party campaigned for a hard/WTO style Brexit, seeking to put pressure on the government during the withdrawal negotiations. In the Brexit Party’s sole EP election campaign, Farage “no longer … [bothered] with criticism of the EU itself” ( Dennison, 2020: 129). The party did however invoke overtly populist themes in its desire to cause a revolution in British politics, change politics for good, and challenge the political establishment ( Tournier-Sol, 2021: 386).

The Brexit Party’s rebrand to Reform UK saw the maintenance of “its Eurosceptic heritage and credentials” and signalled its “evolution into a fully-fledged right-wing populist party, unbound from its single-issue past” ( Hayton, 2025). The party’s populist credentials are clear from the 2024 manifesto, which pledged to “end the corruption of our government and politics by an out-of-touch, London centric elite to make Britain a more democratic, accountable, and … prosperous nation” ( Reform UK, 2024).

On the UK-EU relationship, Reform remained in step with its Eurosceptic origins with plans to scrap retained EU regulations and laws, “abandon the Windsor Framework”, maintain the independence of Britain’s armed forces from EU command structures, and “prepare for renegotiations” of the TCA, to collectively “grasp the huge opportunities of Brexit” (ibid.). Their manifesto implies a preference for increased divergence from the EU, opposition to future - and perhaps deeper - instances of UK-EU co-operation, and dissatisfaction with the post-Brexit arrangements negotiated by the Conservatives (specifically the Windsor Framework and the TCA). This position demonstrates continuity with the Brexit Party, specifically its concerns regarding the Brexit deal.

As mentioned above, UKIP, the Brexit Party, and Reform have mostly operated during distinct periods, with each following the other, at least in terms of their political relevance. This means it is hard to discern the evolution in their positions on Europe in response to recent events the EU has faced such as Brexit, the Covid pandemic, the war in Ukraine, and Donald Trump’s re-election. The persistent hard Euroscepticism and withdrawalist position of the three parties seemingly leaves little room for significant shifts in their position on Europe.

## 4. Responses to Euroscepticism and consequences

The response of the two largest mainstream parties in the UK – the Conservative Party and the Labour Party - to the rise in Euroscepticism has differed. The Conservative Party has a soft (er) Eurosceptic tradition which can be traced back to Margaret Thatcher’s leadership. In the 2000s, the party adopted a soft Eurosceptic position, campaigning to keep the pound in debates over the potential adoption of the euro in Britain and pledging to seek EU reform. This saw the Tories adopt an adversarial strategy towards UKIP ( Lynch and Whitaker, 2013: 299).

The Tories initial response to UKIP’s increased political relevance, coherence, and professionalism in the 2000s was also dismissive. Michael Howard described UKIP as “cranks and political gadflies” in a leaked briefing paper in 2004. Howard’s successor, David Cameron, meanwhile called UKIP supporters “fruitcakes, loonies and closest racists” in 2006. Under David Cameron’s early leadership, the Conservatives approach to the EU focused on offering referendums on specific aspects of integration (see his proposed referendum on the Lisbon Treaty) and seeking further EU reform, whilst retaining British membership.

In response to UKIP’s increased electoral success, the political pressure this brought, and increased division in his party over Europe, Cameron pledged in 2013 to hold an in-out EU referendum if the Conservatives won the 2015 election. This was designed to neutralise the electoral threat posed by UKIP and lance the boil of Euroscepticism. Leading figures in the party variously campaigned for Leave or Remain in the referendum, with Cameron himself leading the Remain campaign, adopting an adversarial stance to Eurosceptic discourse. During the referendum campaign, Cameron acknowledged the EU’s problems but said on balance he was resolutely in favour of staying in and reforming the EU from the inside.

After the referendum, the Conservatives under Theresa May promised to respect the Leave vote, deliver Brexit – “Brexit means Brexit” - and adopted a hardline Eurosceptic position. This, along with the referendum result, crowded out and nullified UKIP, which saw a decline into irrelevance, in line with an accommodative strategy. The Conservatives under Boris Johnson appeared to adopt a more strident approach to delivering EU exit in response to pressure arising from the success of the Brexit Party’s populist Euroscepticism ( Dennison, 2020: 130). After winning the party leadership in 2019, Johnson “set about completing the transformation of the Conservatives into the party of Brexit” ( Hayton, 2025), further claiming ownership of the issue.

The Labour Party has largely been positive towards European integration since the 1990s. The party leadership has been relatively restrained in making the case for the EU in the context of the UK’s wider Eurosceptic political and media climate. During the 2016 referendum, Labour’s leadership supported Remain, adopting an adversarial response to Leave’s Eurosceptic discourse. That said, the vitality of Jeremy Corbyn’s efforts during the campaign has been questioned ( Cole, 2020: 436; Demata, 2019: 127–8). Under Keir Starmer, the party has committed to respecting the Brexit settlement agreed by the Conservatives, whilst pursuing some small measures to further improve and refine aspects of the UK-EU relationship.

The Liberal Democrats, as a determinedly pro-EU party, adopted an adversarial approach in response to Eurosceptic discourse. The Lib Dems campaigned to “stop Brexit” in 2019. Though the party remained relatively quiet on the EU in the period leading up to the 2024 general election.

The growth of populist Euroscepticism in Britain has had wide and concrete consequences for the UK, as well as for the EU. Populist Euroscepticism, particularly expressed by UKIP, helped shift an issue that was at the fringes of British politics to its very centre. Following the Brexit vote, the two traditional mainstream parties (the Conservatives & Labour) have essentially adopted hard Eurosceptic positions, with neither countenancing a return to the EU. Further concrete consequences include the decision to pledge a membership referendum in the first place, arguably the referendum result itself, and the strong version of Brexit that was ultimately delivered (no single market membership). The reluctance to re-open bigger questions regarding the UK-EU relationship, such as a potential return to the single market, means populist Euroscepticism has helped close-off and lock-in certain red lines and profoundly shape British politics for some time to come.

## 5. Short conclusion

The UK represents an especially interesting case study of the intersection of populism and Euroscepticism given its status as the sole country to have left the EU. Britain’s experience of leaving has perhaps served as a warning for other populist Eurosceptic parties in the EU and reduced the viability of them employing an exit strategy (see Van Kessel et al., 2020). As the report has shown, populist Euroscepticism has seen the most success among PRR parties, specifically the parties headed by Nigel Farage. UKIP, although no longer an influential force, effectively fused populism and Euroscepticism in what Usherwood describes as “single issue populism” ( Usherwood, 2019: 1223). UKIP’s successor, the Brexit Party had a short-lived but notable influence on the Conservative party and the Brexit negotiations. The transformation of the Brexit Party into Reform UK has seen it further diversify its electoral offering but retain its scepticism towards the EU and European integration.

## Ethics and consent

Ethical approval and consent were not required.

## Data Availability

No data are associated with this article.

## Footnotes

^1^
George Galloway was also briefly the Workers Party of Britain MP for Rochdale in 2024.
