# Supplementary material for: EUPopLink Country report - United Kingdom
Source: Open Res Eur. 2026 Apr 19;6:126. [Version 1] doi: 10.12688/openreseurope.23498.1 (PMC13133619; doi:10.12688/openreseurope.23498.1)
Supplement: Peer review history

# Reviewer report: Reviewer response for version 1

**DOI:** 10.21956/openreseurope.25437.r72778

**Published:** 2026-05-28

**Authors:** Marianna Griffini, Margherita de Candia

**Affiliations:** 1 Politics and International Relations, Northeastern University London, London, UK; 2 King's College London (Ringgold ID: 4616), London, England, UK

**Brief summary of the article**

 This article provides an overview of populist Eurosceptic parties in the United Kingdom (UK), offering historical insight into the evolution of the UK’s complicated relationship with the European Union (EU). Informative contextual background - notably the role of Euroscepticism in the Labour and Conservative parties - has been provided, alongside in-depth details on the three main populist Eurosceptic parties in the UK. 

**Strengths**

- The article does a good job at providing a general overview of the party scene, showing breadth of knowledge, to which it adds some historical depth.
- The analysis of the Eurosceptic elements in the two mainstream parties, i.e. Labour and Conservatives, highlights dynamics of party competition.
- Some useful electoral data provide an evidence base for the analysis.

**Weaknesses**

- Respect Party: Given the very limited role and relevance of this party, an explicit paragraph on it is perhaps not needed. Otherwise, more details on its links to Euroscepticism need to be provided to justify its inclusion in the current form.
- The article could elaborate more on what makes the UK a ‘special’ case: Namely the fact that Euroscepticism has not been a fringe phenomenon only. A brief mention of the fact that the Labour Party used to be the main Eurosceptic party within the political mainstream, before its conservative rival replaced it in this role, would add informative historical detail to the review (e.g., the 1975 UK’s first referendum on EU membership was called by Labour).
- The article should slightly expand on what it is meant by the ‘turning points’ determining the mainstreaming of Euroscepticism.
- The article should add more details on Reform UK ideological platform, especially regarding the ‘public services’ positions.
- When you mention the Brexit Party platform, you should explain how their campaign messages were distinctly populist: populism seeks change, but broadly speaking all parties aim at changing the political status quo for good. 
- In order to enhance the conceptual framework, the article could add a brief explanation of what single-issue parties are.
- The structure presents room for improvement: It would be useful to streamline it, since sometimes you hint at some points that are then developed much later in the report. One way to do this is by providing all background info (including much of what is currently in Section 4) after the Introduction. The article could also provide all the information on the position on the EU/European integration within each party’s individual paragraphs.

**Minor points: **

- To enhance accuracy, please note that EFD became EFDD (Europe of Freedom and Direct Democracy) in 2015 with the entry of the Five Star Movement. 
- Leave.EU is mentioned in passing; for readers unfamiliar with UK politics, it would be useful to briefly clarify the nature and (short-lived) role of this political organisation. Similarly, a brief explanatory note could be added to clarify what the Windsor Framework is.
- It would be useful, for the sake of completeness, to add a mention to the British National Party, which also campaigned on Euroscepticism, despite being a minor party.

**Editorial suggestions for style/ grammar**

- The article would benefit from a further round of proofreading to smooth out a few unclear sentences. 
- Some references are missing, especially when you mention electoral data and when you mention the Tories’ denigratory comments on UKIP. 
- “The vitality of Jeremy Corbyn’s”: The term ‘vitality’ seems to attribute the shortcoming to Corbyn’s personal energy or character, rather than to the degree of his commitment to the Remain campaign. 

**Concluding section**

- Conclusions could be strengthened by reflecting on what lessons the UK case offers on the intertwining between populism and Euroscepticism.

Is the case presented with sufficient detail to be useful for teaching or other practitioners?

Partly

Is the work clearly and accurately presented and does it cite the current literature?

Partly

If applicable, is the statistical analysis and its interpretation appropriate?

Not applicable

Are all the source data underlying the results available to ensure full reproducibility?

No source data required

Are the conclusions drawn adequately supported by the results?

Yes

Is the background of the case’s history and progression described in sufficient detail?

Partly

Reviewer Expertise:

Marianna Griffini: populism; radical right; gender; party politics in Europe and the US; Margherita de Candia: far right in the European Parliament; UK relationship with the EU

We confirm that we have read this submission and believe that we have an appropriate level of expertise to confirm that it is of an acceptable scientific standard, however we have significant reservations, as outlined above.

# Reviewer report: Reviewer response for version 1

**DOI:** 10.21956/openreseurope.25437.r72782

**Published:** 2026-05-05

**Authors:** Bruno González-Cacheda

**Affiliations:** 1 University of Vigo, Vigo, Spain

This study addresses a relevant topic, but several issues should be addressed to strengthen its analytical clarity and academic contribution:

 1) Lack of a clearly stated objective

 The introduction provides useful contextual background but does not explicitly state the main objective of the study. Clearly articulating the aims of the report would improve the overall coherence of the manuscript and help guide the reader through the analysis.

 2) Conceptual ambiguity regarding populism

 The concept of populism is central to the study, yet the manuscript does not specify the theoretical approach adopted. Given the diversity of definitions in the literature (ex. Cas Mudde; Kurt Weyland), it is important that the author clearly defines and situates their understanding of populism within existing debates. In addition, the concept should be operationalised to clarify how it is applied in the empirical analysis.

 3) Alignment between objectives, analysis and conclusions

 The results and conclusions would benefit from a clearer connection to the (currently implicit) objectives of the study. Moreover, the evidence presented should be more explicitly linked to the chosen definition and operationalisation of populism to ensure analytical consistency.

Is the case presented with sufficient detail to be useful for teaching or other practitioners?

Partly

Is the work clearly and accurately presented and does it cite the current literature?

Partly

If applicable, is the statistical analysis and its interpretation appropriate?

Not applicable

Are all the source data underlying the results available to ensure full reproducibility?

Yes

Are the conclusions drawn adequately supported by the results?

Partly

Is the background of the case’s history and progression described in sufficient detail?

Yes

Reviewer Expertise:

Political Science

I confirm that I have read this submission and believe that I have an appropriate level of expertise to confirm that it is of an acceptable scientific standard, however I have significant reservations, as outlined above.

# Reviewer report: Reviewer response for version 1

**DOI:** 10.21956/openreseurope.25437.r72785

**Published:** 2026-04-30

**Authors:** Vít Hloušek

**Affiliations:** 1 Metropolitan University Prague, Prague, Czech Republic

The paper represents brief but very useful survey of the current link between populism and Euroscepticism in the United Kingdom. My suggestions are not disputing the merit of the paper, which is an accurate and well-structured information.

 I suggest to define both populism and Euroscepticism briefly in the intro. Both concepts are so widely discussed that a reader might not know what exactly does the author mean. I do not suggest to insert a long and heavily theoretical discussion, yet a brief information on what is populism and what is the soft and hard Euroscepticism would be useful.

 My second suggestion would be to tackle briefly populists withing the mainstream parties, especially the Conservatives, such as Boris Johnson. Again, this does not require a long descriptions, yet the mainstreaming of populism and Euroscepticism is an Europe-wide phenomenon and shall be recognised as such also in the British politics.

Is the case presented with sufficient detail to be useful for teaching or other practitioners?

Yes

Is the work clearly and accurately presented and does it cite the current literature?

Yes

If applicable, is the statistical analysis and its interpretation appropriate?

Not applicable

Are all the source data underlying the results available to ensure full reproducibility?

No source data required

Are the conclusions drawn adequately supported by the results?

Yes

Is the background of the case’s history and progression described in sufficient detail?

Yes

Reviewer Expertise:

Party politics, Euroscepticism, Europeanisation, Contemporary European history

I confirm that I have read this submission and believe that I have an appropriate level of expertise to confirm that it is of an acceptable scientific standard.

# Reviewer report: Reviewer response for version 1

**DOI:** 10.21956/openreseurope.25437.r72781

**Published:** 2026-04-23

**Authors:** Sandro Tabatadze

**Affiliations:** 1 Tbilisi State University, Tbilisi, Tbilisi, Georgia

The report presented here provides a well-structured and coherent discussion of the UK case within a limited space. It clearly traces the historical development of Euroscepticism, maps the main populist actors across different periods, and situates their positions within the broader evolution of the UK–EU relationship. The use of secondary literature is consistent and appropriate, supporting the narrative without overloading the text.

 However, several refinements could further strengthen its analytical contribution.

 1. The analysis relies heavily on the soft/hard Euroscepticism typology; incorporating additional distinctions (e.g., strategic vs. principled, sovereignty- vs. policy-based, etc) would enable a more nuanced comparison across cases.

 2. The discussion of party responses could be broadened by including additional actors, such as the Greens, to better capture the full dynamics of the party system.

 3.  The conclusion could more explicitly demonstrate how populism and Euroscepticism reinforce each other in the UK context, rather than leaving this relationship implicit.

Is the case presented with sufficient detail to be useful for teaching or other practitioners?

Yes

Is the work clearly and accurately presented and does it cite the current literature?

Yes

If applicable, is the statistical analysis and its interpretation appropriate?

Not applicable

Are all the source data underlying the results available to ensure full reproducibility?

No source data required

Are the conclusions drawn adequately supported by the results?

Yes

Is the background of the case’s history and progression described in sufficient detail?

Yes

Reviewer Expertise:

Euroscepticism, populism, far right, policy transfer

I confirm that I have read this submission and believe that I have an appropriate level of expertise to confirm that it is of an acceptable scientific standard.
